# Comparison of efficacy and toxicity of intravitreal melphalan formulations for retinoblastoma

**DOI:** 10.1371/journal.pone.0235016

**Published:** 2020-07-01

**Authors:** Terry Hsieh, Albert Liao, Jasmine H. Francis, Jessica A. Lavery, Audrey Mauguen, Scott E. Brodie, David H. Abramson

**Affiliations:** 1 Stein Eye Institute, David Geffen School of Medicine at UCLA, Los Angeles, CA, United States of America; 2 Ophthalmic Oncology Service, Memorial Sloan-Kettering Cancer Center, New York, NY, United States of America; 3 Emory Eye Center, Emory University School of Medicine, Atlanta, GA, United States of America; 4 Department of Ophthalmology, Weill-Cornell Medical Center, New York, NY, United States of America; 5 Department of Epidemiology & Biostatistics, Memorial Sloan-Kettering Cancer Center, New York, NY, United States of America; 6 Department of Ophthalmology, NYU Langone Health, New York, NY, United States of America; Hospital JP Garrahan, ARGENTINA

## Abstract

**Objective:**

Intravitreal melphalan injections are commonly used in the treatment for intraocular retinoblastoma. This study compares retinal toxicity and ocular survival between two formulations, with and without propylene glycol (Alkeran vs. Evomela, respectively).

**Methods:**

A retrospective cohort study of retinoblastoma patients who received intravitreal injections of Alkeran and Evomela at 30 μg from September 2012 to January 2019 at a single tertiary care center were enrolled. Retinal toxicity was measured using electroretinogram (ERG) and compared using a multivariate analysis of 338 injections in 101 eyes of 96 patients. Ocular survival of 163 eyes in 150 patients was compared across formulations using Cox proportional hazards model. Eyes were censored at the time a patient received a dose other than 30 μg.

**Results:**

Overall, ERG decline (mean, 95% CI) for each injection was -5.58 μV (-7.17, -3.99). No significant differences in ERG decrement were found between Alkeran (with alcohol) -5.52uV (-6.99, -4.05). and Evomela (without alcohol) -5.65uV (-8.31 to -2.98) formulations (p = 0.93). Ocular survival at 24 months was 93.6% (95% CI 86.2, 97.1) with alcohol and 91.7% (95% CI 53.9, 98.8) without alcohol. The hazard ratio (HR) for without vs with alcohol was 0.50 (95% CI 0.06 to 4.07); no significant difference in ocular survival was found between formulations (p = 0.52)

**Conclusions and relevance:**

No differences were found in retinal toxicity and ocular survival between 30 μg intravitreal injections of Alkeran or Evomela for intraocular retinoblastoma. Given the increased stability of Evomela, intravitreal treatment could be expanded to centers without the ability to supply Alkeran due to its shorter safety window; however, Alkeran is less expensive. For those with existing infrastructure, Alkeran is a comparable, cost-effective alternative.

## Introduction

Intravitreal melphalan is now used routinely worldwide for treating vitreous seeding in retinoblastoma. It has a high success rate without increased risk of extraocular extension or metastases [1–3]. Previous work demonstrates that melphalan, an unstable alkylating agent, works through direct anti-tumor and anti-angiogenic properties [4].While intravitreal (unlike intravenous) melphalan has very limited systemic toxicity, it does have intraocular toxicity which may involve the retina, lens, cornea and iris^,^[5]. Melphalan, is thought to cause collateral toxicity via direct effect, degradation into harmful metabolites, side effect of its vehicle, or consequence of ocular penetration.

Two forms of injectable melphalan are now available: Alkeran and Evomela. Alkeran is melphalan reconstituted with a proprietary diluent containing sodium citrate, ethanol, povidone, and propylene glycol (PG) [6]. Previous studies have shown that reconstituted Alkeran (hereafter “with alcohol” or propylene glycol melphalan, PGM) degrades to less than 95% of initial concentration after 1–2 hours [7]. However, PG has been associated with systemic toxicity when given intravenously, with reactions such as hyperosmolality, lactic acidosis, acute kidney failure, and systemic inflammatory response syndrome [8]. Although the mechanism of PG toxicity is unknown, intravitreal PG or the ethanol component could exacerbate melphalan’s inherent toxicity. Evomela (hereafter “without alcohol” or Captisol-stabilized melphalan, CSM), was approved in 2016 as melphalan with Captisol (a beta-cyclodextrin derivative) reconstituted in normal saline, to improve the stability and solubility of melphalan, as well as reduce systemic toxicity [9]. CSM has demonstrated superior stability after reconstitution, usable up to 4–5 hours and significantly reduced degradation into toxic metabolites [10].

Our center switched in November 2016 from the PGM (Alkeran, Apopharma USA Inc.) to CSM (Evomela, Acrotech Biopharma LLC) with the hope that it would decrease retinal toxicity while retaining efficacy for treatment of intraocular retinoblastoma. This study compares the degree of retinal toxicity and overall ocular survival between formulations in a large, single-center retrospective analysis.

## Materials and methods

This retrospective study was approved by the Institutional Review Board from Memorial Sloan Kettering Cancer Center. All eyes that received injections of 30 μg of melphalan for intraocular retinoblastoma between September 2012 and January 2019 were included for the analysis. All patient data was collected from Memorial Sloan Kettering Cancer Center. Patients that changed dosage and subsequently returned to 30 μg were excluded (n = 3). All patients had greater than 2 months follow up from their first injection. Informed consent for treatment was obtained for each patient by their parent, guardian, or caregiver. A waiver of informed consent for use of medical records for this retrospective research protocol was granted by the Institutional Review Board. This study is compliant with the Health Insurance Portability and Accountability Act (HIPAA).

Of 353 injections to 103 eyes in 97 patients, injections missing ERG information due to the absence of electrophysiologist were excluded, leaving 338 injections to 101 eyes in 96 patients eligible for analyses of retinal toxicity. Data was analyzed as follows: for ERG measurements, a multivariable repeated measures linear model with a random intercept and slope, accounting for repeated measures to each eye was implemented (eyes to the same patient were considered independent). The fixed effects in this model were injection number, formulation (PGM, CSM) and an interaction term between injection number and formulation to assess whether the change in ERG over time varies by formulation. We examined the univariable association of ERG with age (at injection), weight (at baseline), iris color, concomitant topotecan, concomitant ophthalmic artery chemosurgery (OAC), concomitant focal treatment, time (weeks) between injections, and new injection clock site hour. Variables significant in univariable analyses at p<0.20 were included in a multivariable model.

Ocular survival was defined as the time from the first injection to enucleation. Eyes were censored at the time the patient received a dose other than 30 μg. Eyes that were not enucleated were censored at the end of follow-up. Ocular survival was visualized using Kaplan-Meier methods based on the eye’s initial injection formulation. For patients with last known follow up and injection on same day, one day was added to include them in analysis. Ocular survival was analyzed formally by a Cox proportional hazards model for 163 eyes in 150 patients with a time-dependent covariate to account for changing formulation over time. Given the retrospective nature of the study, a power calculation was not performed. The sample size is based on all patients who were eligible for analysis given the rarity of the disease. Analyses were performed in SAS v9.4.

## Results

Baseline characteristics are shown for patients analyzed in the ERG dataset (Table 1). Although more patients treated with PGM had more advanced disease by Reese-Ellsworth criteria, the two groups are similar by International Classification of Retinoblastoma (ICRB-COG scheme). The PGM treatment group had a larger percentage of concomitant OAC and topotecan treatment compared to CSM, while the CSM had a larger percentage of focal treatment (either laser or cryotherapy).

**Table 1:** Patient demographics by formulation.

Characteristics	30ug (PGM, with alcohol) n (%)	30ug (CSM, without alcohol) n (%)
Number of injections	255	98
Number of eyes ^a^	70	41
*Eye*		
OD	127 (49.8)	36 (36.7)
OS	128 (50.2)	62 (63.3)
*Age at injection (years)*		
Median (range)	3 (0, 18)	3 (1, 16)
*Weight (kg) at baseline*		
Median (range)	14 (7, 63)	15 (5, 62)
*Reese-Ellsworth Classification*		
Unknown	231	0
2A	0 (0.0)	2 (2.0)
2B	0 (0.0)	1 (1.0)
3A	2 (8.3)	11 (11.2)
3B	0 (0.0)	5 (5.1)
4A	0 (0.0)	8 (8.2)
4B	0 (0.0)	2 (2.0)
5A	0 (0.0)	7 (7.1)
5B	22 (91.7)	62 (63.3)
*International Classification of Retinoblastoma (ICRB)*		
Unknown	231	0
A	0 (0.0)	1 (1.0)
B	1 (4.2)	6 (6.1)
C	1 (4.2)	3 (3.1)
D	18 (75.0)	75 (76.5)
E	4 (16.7)	13 (13.3)
*Time between injections*		
Unknown	0	6
No prior injections	67 (26.3)	33 (35.9)
< = 1 week	114 (44.7)	0 (0.0)
1–2 weeks	16 (6.3)	3 (3.3)
2–4 weeks	3 (1.2)	30 (32.6)
4+ weeks	55 (21.6)	26 (28.3)
*Iris color*		
Blue	33 (12.9)	32 (32.7)
Light brown	85 (33.3)	11 (11.2)
Dark brown	137 (53.7)	55 (56.1)
*Seed type*		
Non-vitreous	54 (21.2)	34 (34.7)
Vitreous	201 (78.8)	64 (65.3)
*Concomitant OAC*		
No	197 (77.3)	88 (89.8)
Yes	58 (22.7)	10 (10.2)
*Concomitant Topotecan*		
No	211 (82.7)	93 (94.9)
Yes	44 (17.3)	5 (5.1)
*Concomitant focal treatment*		
No	213 (83.5)	11 (11.2)
Yes	42 (16.5)	87 (88.8)
*New injection clock hour* ^b^		
Unknown	9	8
No	168 (68.3)	75 (83.3)
Yes	78 (31.7)	15 (16.7)

OD: right eye

OS: left eye

OAC: ophthalmic artery chemosurgery

^a^ The same eye could have received 25ug and 30ug injections. Therefore, the sum of the number of eyes from the two columns does not equal the number of eyes in the dataset.

^b^ New injection clock site hour was set to “No” for the 1^st^ injection.

Electroretinogram responses were used as a noninvasive measurement of retinal toxicity. Using a multivariable repeated measures linear model (Tables 2 and 3), each injection was associated with a mean (95% CI) decline in ERG of -5.58uV (-7.17, -3.99). PGM injections resulted in a mean decrement of -5.52uV (-6.99, -4.05), similar to CSM at -5.65uV (-8.31 to -2.98) per injection. No significant difference in retinal toxicity was found between formulations (p = 0.93).

**Table 2:** Parameters used in multivariable analysis of ERG degradation over time.

Parameter	Average Change in ERG (95% Confidence Interval)	p-value
**Intercept**	53.61 (36.23, 70.99)	-
**Injection number**	-5.65 (-8.31, -2.98)	< .01
**Formulation**		0.57
**PGM (with alcohol) vs CSM (without alcohol)**	3.63 (-8.83, 16.09)	
Injection number*formulation	0.13 (-2.76, 3.01)	0.93
**Age (at injection)**	1.00 (-3.37, 5.38)	0.65
**Weight (at baseline)**	0.35 (-0.81, 1.51)	0.55
**Iris color**		0.09
**Blue vs light brown**	1.93 (-15.76, 19.61)	
**Dark brown**	-12.18 (-26.32, 1.96)	

**Table 3:** Results from multivariable analysis of ERG degradation over time.

Estimate	Average change in ERG (95% Confidence Interval)
**Overall decline in ERG**	-5.58 (-7.17, -3.99)
**CSM (without alcohol) over time**	-5.65 (-8.31, -2.98)
**PGM (with alcohol) over time**	-5.52 (-6.99, -4.05)

**Fig 1:**
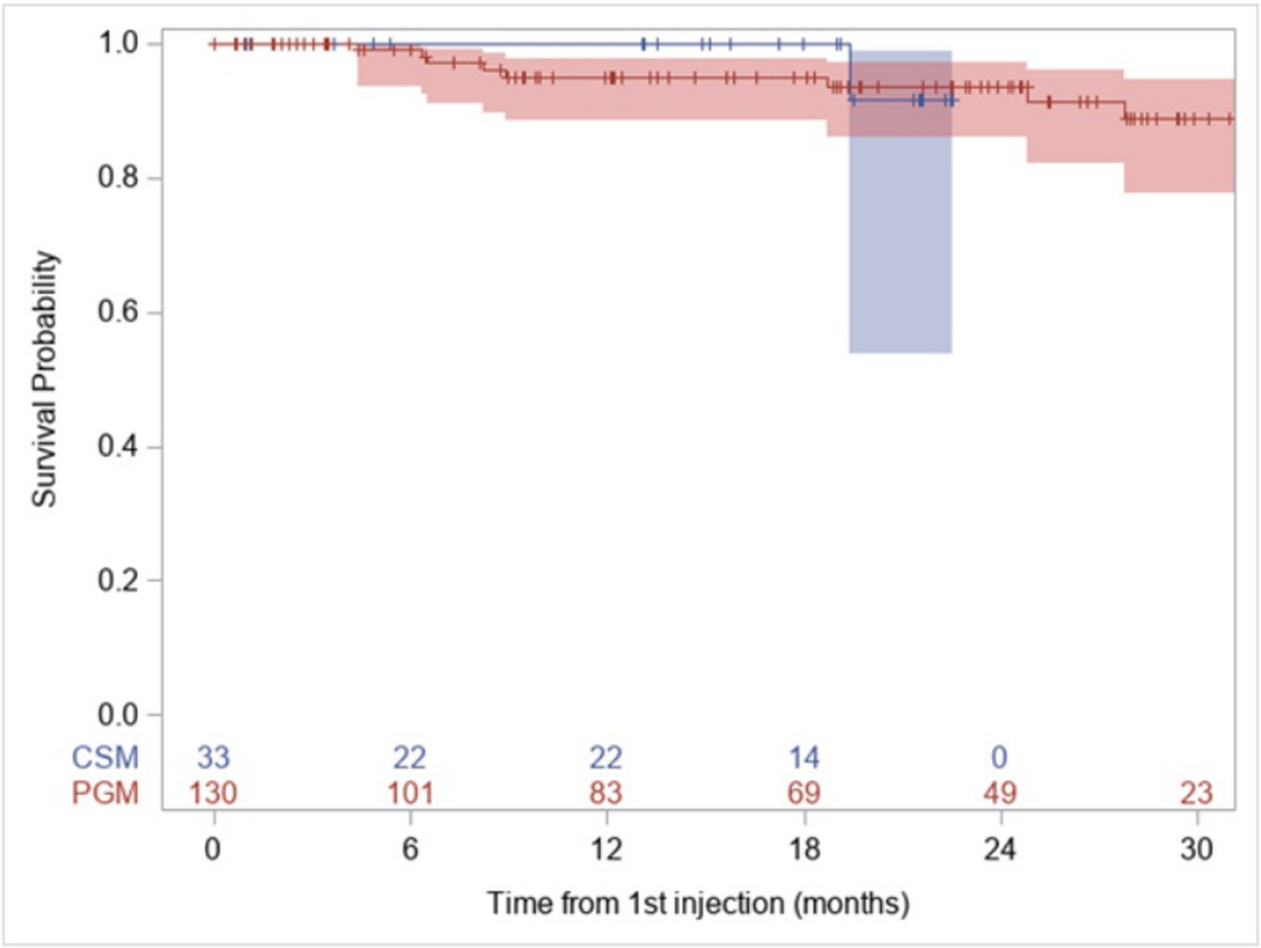
Ocular survival based on formulation of first injection. Kaplan-Meier plot of ocular survival. 9 total enucleations occurred– 8 in PGM group and 1 in CSM group. For the Kaplan-Meier figure, groups are determined by their initial injection. No significant difference was found between formulation and ocular survival (p = 0.50) and hazard ratio for CSM (without alcohol) to PGM (with alcohol) is 0.50 (95% CI 0.06, 4.07).

Ocular survival is an important metric to determine the efficacy of intravitreal injection in limiting intraocular progression and extraocular extension (Fig 1). At 12 months, the PGM group had 95% (95% CI 88.5, 97.9) ocular survival compared to 100% in the CSM group (based on the first injection, Table 4). 24-month ocular survival was similar with PGM survival at 93.6% (95% CI 86.2, 97.1) compared to eyes receiving CSM at 91.7% (95% CI 53.9, 98.8). A total of 9 eyes were enucleated in this set, 8 from PGM, 1 from CSM. Additionally, out of the 16 eyes that were censored at the time of receiving a dose other than 30ug, six eyes were eventually enucleated. These eyes were censored in the analysis of ocular survival when they crossed over into a lower dose injection because their enucleation could not be exclusively attributed to the injection formulation. The hazard ratio comparing CSM to PGM was 0.50 (0.06, 4.07, p = 0.52).

**Table 4:** Comparison of ocular survival between formulations.

Formulation	12-month survival (95% CI) *	24-month survival (95% CI) *
**PGM**	95.0% (88.5, 97.9)	93.6% (86.2, 97.1)
**CSM**	100%	91.7% (53.9, 98.8)

*Based on a patient’s first injection.

## Discussion

Intravitreal chemotherapy is an effective treatment for vitreous seeding in retinoblastoma, but it comes at the expense of irreversible toxicity [1]. With the approval of Evomela in 2016, our group switched from Alkeran, hoping that the elimination of propylene glycol and ethanol would decrease retinal toxicity. This study demonstrates no significant differences between formulations in retinal toxicity or ocular survival.

Our data is relevant to the current healthcare dilemma of providing access to treatments in the setting of rapidly increasing chemotherapy costs. At our institution, Evomela is four-fold more expensive than Alkeran [11, 12], but has longer stability than Alkeran. Given the findings of this study, centers that have the infrastructure to deliver Alkeran within its short stability window could continue using it to save healthcare and patient costs with no differences in outcomes. On the other hand, using Evomela, with its longer stability window could expand access to intravitreal chemotherapy to hospitals and treatment centers without that infrastructure, albeit at an increased price.

Strengths of this study include the large dataset of patients and longitudinal objective measurements of retinal toxicity via ERG. Furthermore, the degree of retinal toxicity from melphalan was consistent with our previously published data [13]. Weaknesses of this study include its retrospective nature, whereby patients were not randomized into groups. However, patients were treated consecutively after the formulation change without additional selection criteria. Accordingly, patients in each group reflect trends in treatment, as Evomela group patients were treated using more focal methods instead of topotecan. The low number of enucleations prevented us from adjusting for potential confounders such as Reese-Ellsworth criteria and concomitant treatments, limiting the inferences that can be drawn from this comparison.

## Conclusions

This study did not find evidence of a difference between Evomela and Alkeran formulations of melphalan with respect to retinal toxicity and ocular survival, allowing centers the flexibility to choose between the two formulations based on cost and stability needs. This may expand access to care for eligible patients.

## Supporting information

S1 Table Data set used for analysis of ERG degradation over time and survival analysis. (XLSX)
